# In vitro bioprocessing of corn as poultry feed additive by the influence of carbohydrate hydrolyzing metagenome derived enzyme cocktail

**DOI:** 10.1038/s41598-021-04103-z

**Published:** 2022-01-10

**Authors:** Seyed Hossein Mousavi, Seyedeh Fatemeh Sadeghian Motahar, Maryam Salami, Kaveh Kavousi, Atefeh Sheykh Abdollahzadeh Mamaghani, Shohreh Ariaeenejad, Ghasem Hosseini Salekdeh

**Affiliations:** 1 Department of Systems and Synthetic Biology, Agricultural Biotechnology Research Institute of Iran (ABRII), Agricultural Research Education and Extension Organization (AREEO), P. O. Box, 31535-1897 Karaj, Iran; 2 Department of Food Science and Engineering, University College of Agriculture & Natural Resources, University of Tehran, Karaj, Iran; 3 Laboratory of Complex Biological Systems and Bioinformatics (CBB), Department of Bioinformatics, Institute of Biochemistry and Biophysics (IBB), University of Tehran, Tehran, Iran; 4 Departmen of Molecular Sciences, Macquarie University, Sydney, NSW Australia

**Keywords:** Biochemistry, Biotechnology, Computational biology and bioinformatics, Microbiology, Molecular biology, Structural biology

## Abstract

The carbohydrate-hydrolyzing enzymes play a crucial role in increasing the phenolic content and nutritional properties of polysaccharides substrate, essential for cost-effective industrial applications. Also, improving the feed efficiency of poultry is essential to achieve significant economic benefits. The current study introduced a novel thermostable metagenome-derived xylanase named PersiXyn8 and investigated its synergistic effect with previously reported α-amylase (PersiAmy3) to enhance poultry feed utilization. The potential of the enzyme cocktail in the degradation of poultry feed was analyzed and showed 346.73 mg/g poultry feed reducing sugar after 72 h of hydrolysis. Next, the impact of solid-state fermentation on corn quality was investigated in the presence and absence of enzymes. The phenolic content increased from 36.60 mg/g GAE in control sample to 68.23 mg/g in the presence of enzymes. In addition, the enzyme-treated sample showed the highest reducing power OD 700 of 0.217 and the most potent radical scavenging activity against ABTS (40.36%) and DPPH (45.21%) radicals. Moreover, the protein and ash contents of the fermented corn increased by 4.88% and 6.46%, respectively. These results confirmed the potential of the carbohydrate-hydrolyzing enzymes cocktail as a low-cost treatment for improving the phenolic content, antioxidant activity, and nutritional values of corn for supplementation of corn-based poultry feed.

## Introduction

Cellulose, hemicelluloses, and lignin, are the main source of fermentable sugars in lignocellulosic feedstock^1^. Carbohydrate-active enzymes are biocatalysts capable of converting and modifying the polysaccharides substrate for several industrial purposes, particularly in biorefinery and food applications^2^. These enzymes are mostly responsible for the release of functional compounds such as phenolics during fermentation^3,4^. Phenolic compounds are essential phytochemicals found in plants that have been linked to reducing the risk of several chronic diseases, mostly due to their high potential in radical scavenging and chelating metals^5^. Due to the antioxidant potential of corn, it can be considered as a capable grain for the production of animal feeds^6^. This crop is one of the main ingredients in poultry and animal diets^7^; therefore, using feasible strategies to improve the quality of this ingredient is essential for supplementation of animal feed^8^.

Solid-state fermentation (SSF) is an advantageous process used to enhance the functional properties of crops. This environmentally friendly, economical technique produces bioactive compounds from different agricultural residues^9^. Moreover, fermentation is reported as a tool that can increase the antioxidant and bioactive potential of plant material^10^. SSF process is widely used to improve animal and poultry feed quality^11^. Furthermore, SSF coupled with carbohydrate-active enzymes was proposed as a potential strategy for feed quality improvement^12,13^.

Among the various carbohydrate-hydrolyzing enzymes, xylanase and $\mathrm{\alpha }$-amylase proved their potential in numerous industrial fields, especially in animal feed. Xylanase and $\mathrm{\alpha }$-amylase are derived from plants, animals and microorganisms, and cleave the β-1,4 linkages of hemicelluloses in the plant cell wall and α-1,4-glycosidic bonds in starch, respectively^14,15^. Various techniques can be used for enhancing enzymatic activity. Enzyme immobilization and designing carboxymethyl cellulose (CMC)-based hydrogel carriers reported as a powerful tool for improving the operational stability, reusability, pH tolerance and thermal stability of xylanases^16–18^. Also, the production of the enzyme cocktail and the addition of the compatible solutes can effectively increase the activity and stability of enzymes^16,19^. Identification of the thermostable xylanase and $\mathrm{\alpha }$-amylase is essential for the poultry industry. The metagenomic tool has evolved to discover the novel carbohydrate-hydrolyzing enzymes from the uncultivable component of microbial communities^20^. Besides, the in-silico screening strategy and computationally assisted methodology were reported as a powerful method for the identification of several improved enzymes for industrial applications^21–24^. This pipeline was used to identify the novel potential xylanases and amylases for effective degradation of raw materials^25,26^.

In this context, the present study developed an eco-friendly process and efficient enzyme cocktail to improve the bioactive and nutritional properties of corn. The strategy was to produce a carbohydrate-active enzyme cocktail capable of degrading the poultry feed using novel thermostable xylanase and also previously reported thermostable α-amylase^26^. The change in the phenolic content, antioxidant capacity, and nutritional value of the corn were investigated for its applicability in improving poultry feed nutrition and supplementation of corn-based diets.

## Materials and methods

### Raw materials, chemicals and microorganism strain

All chemicals including 3,5-Dinitrosalicylic acid (DNS), Bovine serum albumin (BSA), beechwood xylan, Na_2_CO_3_, avicel, $\upbeta$-glucan, xylan, filter paper, locust bean gum (LBG), carboxymethylcellulose (CMC), coomassie brilliant blue, ethanol, phosphoric acid, metal ions, ethylenediaminetetra acetic acid (EDTA), urea, phenylmethylsulfonyl fluoride (PMSF), sodium dodecyl sulfate (SDS), cetrimonium bromide (CTAB), Tween 20, Triton X-100, NaN_3_, HCl, NaOH, beta mercaptoethanol, methanol, acrylamide, bisacrylamide, temed, ammonium persulfate (APS), glycine, tris-base, 2,2'-azino-bis(3-ethylbenzothiazoline-6-sulfonic acid) (ABTS), 2,2-diphenyl-1-picrylhydrazyl (DPPH), potassium ferricyanide, trichloroacetic acid, ferric chloride, Folin and gallic acid were from Sigma-Aldrich. Corn flour was purchased from local market (Karaj, Iran). The *Saccharomyces cerevisiae* IBRC-M 30069 was purchased from the Iranian Biological Resource Center. To obtain inoculum for SSF, the selected fermentative microorganism, *Saccharomyces cerevisiae *was grown in 250 mL flasks (pH 6.8) and medium containing 0.75 g yeast extract, 0.75 g malt extract, 1.25 g soy peptone, and 1.25 g glucose at 28 °C in a rotary shaker 180 rpm for 24 h until the absorbance at 600 nm reached to approximately 1.2.

Poultry feed gained from the Nican Dam Arvin. The α-amylase (PersiAmy3) was cloned, expressed, purified and characterized according to the previous study^26^. Luria–Bertani medium (LB broth), T4 DNA ligase (Thermo Fisher Scientific), Kanamycin (Duchfa), Isopropyl β-D-1-thiogalactopyranoside (IPTG), *BamH*I and *Sal*I restriction enzyme (Thermo Fisher Scientific), Gel Extraction kit (Thermo Scientific), Ni–NTA Fast Start Kit (Qiagen, Hilden, Germany) were used for production, expression, and purification of xylanase (PersiXyn8). The phosphate buffer with appropriate pH and distilled water were used to prepar the samples.

### In-silico identification, cloning, expression and purification of the novel PersiXyn8

In this study we used, raw cattle rumen Metagenomic data submitted to NCBI with Bio-project ID: PRJNA631951^27^. The cattle rumen metagenomic data was mined to discover a thermostable xylanase enzyme. The quality of metagenomic data was verified by FASTQC and the high-quality data was assembled using meta-velvet assembler. For targeted screening of the assembled contigs, first, the xylanase genes were predicted using MetaGeneMark. Then, TAXyl (Thermal Activity Prediction for Xylanase)^28^ as a machine learning assisted web-based tool which employs supervised algorithms to predict the thermal activity of GH10 and GH11 xylanases was applied on the predicted putative xylanases. TAXyl uses sequence-based and length-independent protein descriptors to predict the thermal activity of xylanases in one of the non-thermophilic, thermophilic, and hyper-thermophilic classes. Considering the application in poultry feed industry, one of thermophile GH10 xylanases predicted by TAXyl, was selected for the next steps. For further confirmation, the NCBI Conserved Domains Database (CDD)^29^ was employed to identify the xylanase domain in predicate xylanase, PersiXyn8. Also, the Phyre2 server^30^ was used to confirm the similarity of the tertiary structure of PersiXyn8 with GH 10 xylanase enzymes. Finally, PersiXyn8 sequence data was deposited in the GenBank under accession number MW349589.

To ensure that PersiXyn8 is a novel xylanase enzyme, we aligned it against NCBI non-redundant protein sequences database using multiple sequence alignments with CLUSTALW (Supplementary Fig. 1).

The ORF coding of xylanase gene was acquired from the cattle rumen metagenome DNA with a pair of forward (5'- TGATAGGGATCCATGAATGAATGGGAAAAGGAAT-3’) and reverse (5'- TGATAGGTCGACTCAGCGTATACTACTGAATC-3’) primers which contain *BamH*I and *Sal*I restriction sites, respectively. The gradient PCR protocol performed as follows: initial denaturation at 95 °C for 5 min; 10 cycles of denaturation at 95 °C for 40 s, gradient annealing at 60 °C to 50 °C for 40 s, and elongation at 72 °C for 90 s and after that 35cycles of denaturation at 95 °C for 40 s, annealing 54 °C for 40 s, and elongation at 72 °C for 90 s. The final cycle was followed by an extension at 72 °C for 10 min. For gel extraction of xylanase DNA fragments, the PCR product was run on the 1% (w/v) agarose gel and DNA fragments was cut with a razor blade and purified then using the GeneJET Gel Extraction Kit (Thermo Fisher Scientific). Further, the extracted fragment was ligated with T/A cloning vector pTZ57/RT and transformed into competent *E. coli* DH5a cells. For the expression of xylanase gene, *BamH*I and *Sal*I digested purified from agarose gel and was ligated to the downstream of the 6 × His tag of similarly digested pET-28a (+) vector and transformed into *E. coli* BL21 (DE3) by heat shock method. Transformants were selected on LB- kanamycin (50 μg/mL) agar plates and confirmed by colony PCR. For expression and purification of the recombinant xylanase, 250 mL of Luria-Bertoni (LB) broth containing 50 μg.mL^−1^ kanamycin was inoculated with 10 mL of overnight grown culture of *E. coli* BL21 (DE3) containing pET-28a-xylanas and was grown in the shaker incubator at 37 °C/180 rpm. When the optical density (OD) of the cell culture reached 0.6 at 600 nm, Isopropyl β-D-1-thiogalactoside (IPTG) (0.25 mM) was added for 18 h at 25 °C in order to xylanase protein induction. The N-terminal Histidine-tagged recombinant protein was purified by Ni–NTA Fast Start Kit (Qiagen, Hilden, Germany) with some modification. Cell suspension was harvested by centrifugation at 8000 × *g* for 10 min and resuspend the cells pellet in 10 mL native Lysis Buffer supplemented with PMSF and DTT and incubated on ice for 30 min after that sonication was down (60% amplitude of 5*1 min with 1 min interval), centrifuge lysate at 14,000 × *g* for 30 min at 4 °C to pellet the cellular debris. Apply the cell lysate supernatant to the Ni–NTA column and 2 times column washed with wash buffer. Elute bound 6xHis-tagged protein with 1 mL aliquots of Native Elution Buffer (50 mM NaH_2_PO_4_, 300 mM NaCl, 250 mM imidazole; pH 8.0). Protein concentration was measured by the method of Bradford^31^.

### Xylanase activity assay

The activity of the xylanase was investigated by preparation of the beechwood xylan in the concentration of 1% (w/v) in 50 mM phosphate buffer, pH 6.0. The reaction mixture composed of 20 $\mathrm{\mu L}$ of the PersiXyn8 (1 mg/mL) and 60 $\mathrm{\mu L}$ of xylan was incubated at 50 $^\circ{\rm C}$ for 20 min. The amounts of released reducing sugars were measured based on the DNS assay^32^. In this connection, 180 $\mathrm{\mu L}$ of DNS reagent was added to the samples and boiled for 5 min. The absorbance was recorded at 540 nm. One unit of the xylanase activity was defined as the amount of enzyme that liberates 1 μmol of reducing sugar per minute based on the xylose standard curve. The specific activity of the enzyme was defined as unit per mg of protein which was estimated according to the Bradford procedure^31^.

### Influence of temperature, pH, metal ions and inhibitors

The optimum temperature of the xylanase activity was studied by incubating the enzyme with beechwood xylan (1% w/v) in phosphate buffer (50 mM, pH 7.0) in a temperature range of 30 to 80 $^\circ{\rm C}$ for 20 min. By fixing the optimum temperature for enzyme, the influence of pH on the xylanase activity was examined by the incubation of PersiXyn8 with the substrate (1% w/v) in 50 mM sodium citrate buffer (pH 4.0–5.0), 50 mM potassium phosphate buffer (pH 6.0–8.0) and 50 mM carbonate-bicarbonate buffer (pH 9.0) at 50 $^\circ{\rm C}$ for 20 min. The xylanase activity was investigated using DNS method, and the relative enzymatic activity was measured by expressing the maximum activity as 100%.

To analyze thermal stability, the PersiXyn8 was evaluated by incubating the enzyme for 120 min at temperatures 40 to 80 ℃ at optimum pH (pH 6.0). The storage stability of the enzyme was estimated by incubating the PersiXyn8 for 8 h at 50 °C. The enzymatic activity was measured at 30 min intervals under mentioned reaction condition.

The enzymatic activity was measured at the presence of 5 mM MgCl_2_, CaCl_2_, NaCl, MnCl_2_, CuSO_4_, FeSO_4_, ZnCl_2_, EDTA, Urea, PMSF and NaN_3_ and 1% SDS, CTAB, Tween 20 and Triton X-100. The enzyme was preincubated with each denaturant for 30 min at room temperature. The substrate (1% w/v) was added to the mixtures and incubated in optimum condition for 20 min. The DNS method was used for determining the amounts of generated reducing sugars, and the relative xylanase activities were investigated. The activity found in the absence of chemicals was taken as control (100%).

### Determination of kinetic parameters

To measur the kinetic parameters of the enzyme, it was incubated in various concentrations of the beechwood xylan from 0.001 mM to 0.04 mM in potassium phosphate buffer (pH 6.0, 50 mM) at 50 °C. The enzymatic activity was determined according to the DNS method and K_m_, K_cat_ and K_cat_/K_m_ of the xylanase were calculated based on the Lineweaver–Burk plot.

### Substrate spectrum of the PersiXyn8

Using different substrates, including avicel, $\upbeta$-glucan, beechwood xylan, filter paper, LBG and CMC, the substrate specificity of the PersiXyn8 was analyzed. Toward that end, the substrates were prepared at the concentration of 1% in phosphate buffer (50 mM, pH 6.0) and incubated with the enzyme (1 mg/mL) at 50 $^\circ{\rm C}$ for 20 min. The amounts of reducing sugars were examined according to the DNS method^33^. One unit of the xylanase activity was defined as the amounts of enzyme that liberated 1 μmol of reducing sugar per minute and the specific activity was expressed as unit per mg of protein.

For thin layer chromatography (TLC) analysis, the PersiXyn8 was incubated with 10 mL of beechwood xylan (0.5 g in 50 mM phosphate buffer pH 9.0) followed by incubation at 50 °C for 12 h. The hydrolyzed products were separated and detected by TLC and D-xylose was used as the reference standard. The mobile phase was a mixture of chloroform/acetic acid/water (6:7:1 by volume). To detect sugar spots, TLC plate was sprayed by solution of 5% H_2_SO_4_ and 95% ethanol and dried the plate at 105 °C for 10 min^34^.

### Developing the carbohydrate-hydrolyzing enzyme cocktail capable of degrading the poultry feed

To investigate the synergistic relationship between the PersiXyn8 (322 U/mg protein) and the reported α-amylase, PersiAmy3 (65 U/mg protein), 20 μL of enzyme mixture (PersiXyn8:PersiAmy3) in various ratios of 100:0, 80:20, 60:40, 40:60, 20:80, 0:100 was added to the 60 μL of poultry feed (20 mg/mL) in 50 mM phosphate buffer (pH 6.0) and incubated at 50 $^\circ{\rm C}$ for 20 min. The DNS method was used for enzymatic activity estimation and the relative activities were measured by taking the maximum activity as 100%.

To study the ability of the enzyme cocktail to hydrolyze the poultry feed, 20 μL of enzyme cocktail in an optimum ratio of 20:80 (PersiXyn8:PersiAmy3, 10 U/g) was added to the 60 μL of poultry feed (20 mg/mL) in 50 mM phosphate buffer (pH 6.0) and incubated at 50 $^\circ{\rm C}$ for 72 h. Samples were taken in 24 h intervals followed by the reducing sugars estimation via DNS method. The sample without enzyme addition was used as a control.

### Solid-state fermentation of corn and enzyme cocktail treatment

Ingredients and nutrients of poultry feed for this study are given in supplementary Table.1. Due to the high percentage of corn in this substrate, it was selected for SSF in the presence of a thermostable enzyme cocktail. Corn flour was sieved through a 50 mesh and sterilized by autoclaving at 110 °C for 10 min. The SSF was processed in the presence of 10 g corn flour, 50 mL medium, active yeast (5%) and enzyme cocktail at the optimum ratio of 20:80 (PersiXyn8:PersiAmy3, 10 U/g) under 80% humidity for 7 days at 28 $^\circ{\rm C}$. The sample without enzyme cocktail and inoculation was used as a control. The fermented materials were lyophilized for 24 h and stored at 4 °C for further analysis.

### Scanning electron microscopy (SEM) analysis

The morphological changes of the corn during SSF and enzyme cocktail treatment was investigated by scanning electron microscopy (SEM). The fermented and control (without enzyme cocktail and yeast) samples were lyophilized for 24 h and their structural changes were analyzed using a scanning electron microscope (FEI Quanta 200, USA) at 15 kV.

### Extraction and determination of total polyphenolic composition

The phenolic compounds of the fermented samples were extracted according to the described method with some modifications^35^. In brief, 2 g of each lyophilized sample was extracted in 40 mL solution of methanol 95% and HCl 1 N (85:15, v/v) for 2 h at room temperature. Later, the solutions were centrifuged at 2500 *g* for 10 min and the supernatants were obtained. Next, the residues from the centrifugation were re-extracted using the methanol 95% and HCl 1 N (85:15, v/v) after 2 h of stirred condition at room temperature followed by the second centrifugation at 2500 *g* for 10 min. The final supernatants were mixed, concentrated under a vacuum at 45 $^\circ{\rm C}$ and stored in the freezer until use. The Folin-Ciocalteu colorimetric method was used to examine the total phenol content of samples^36^. Therefore, 500 μL of Folin reagent 10% was added to 100 μL of diluted sample and mixed with 400 μL of Na_2_CO_3_ 7.5%. The reaction mixture was equilibrated at room temperature for 30 min and the absorbance was recorded at 750 nm. Different concentrations of gallic acid (5–85 μg/mL) were used to plot the standard curve and the phenolic concentration of the sample expressed as the mg of gallic acid equivalents per gram of sample dry weight (mg GAE/g sample).

### Determination of the antioxidant capacity

- **ABTS radical scavenging activity**
  The ability of the fermented samples (1 mg/mL) to scavenge the radical ABTS was performed based on the previous method^37^. At first, the stock solution of 7.4 mM ABTS was mixed with 2.6 mM potassium persulfate solution at the ratio of 1:1 in 50 mM phosphate buffer saline (PBS, pH7.4) and kept in a dark place at room temperature for 12–16 h. Afterwards, the ABTS^+^ solution was diluted with distilled water to reach an absorbance of 0.70 ± 0.02 at 734 nm. Next, 1 mL of ABTS^+^ solution was added to 100 μL of diluted sample or distilled water (control) and mixed by vortexing. The reaction mixture was proceeded at 25 °C for 6 min and the absorbance was read at 734 nm using spectrophotometer. Radical scavenging activity was calculated using the following equation:
  $$\mathrm{\%ABTS scavenging}=\frac{{\mathrm{Abs}}_{\mathrm{control}}-{\mathrm{Abs}}_{\mathrm{sample}}}{{\mathrm{Abs}}_{\mathrm{control}}}\times 100$$
- **DPPH radical scavenging activity**
  The ability of the samples (1 mg/mL) to eliminate the DPPH free radicals was assessed according to the procedure described^38^. Briefly, 1 mL of a freshly prepared ethanolic solution of DPPH (0.1 mM) mixed with 100 μL of diluted sample and shook vigorously. The distilled water was used as a control, after incubating the solution stand in the dark at room temperature for 30 min. The absorbance was measured at 517 nm against a methanol blank. DPPH radical scavenging activity was calculated using the following equation:
  $$\mathrm{\%DPPH scavenging}=\frac{{\mathrm{Abs}}_{\mathrm{control}}-{\mathrm{Abs}}_{\mathrm{sample}}}{{\mathrm{Abs}}_{\mathrm{control}}}\times 100$$
- **Reducing power assay**

The reducing power was measured according to the method described before with some modification^39^. For analysis, 0.5 mL of diluted sample (1 mg/mL) was mixed with 1.25 mL phosphate buffer (200 mM/L, pH 6.6) and 1.25 mL potassium ferricyanide (1% w/v). After incubation for 20 min in a water bath at 50 °C, 1.25 mL trichloroacetic acid (10% w/v) was added to the mixture. The resulting solutions were centrifuged for 10 min at 1500 g. Then 1.25 mL of the supernatants were mixed with 1.25 mL distilled water and 0.25 mL ferric chloride (0.1% w/v). The absorbance was measured at 700 nm after 10 min incubating at room temperature using a UV visible spectrophotometer.

### Determination of protein, lipid and ash contents

Total crude protein was evaluated using the Kjeldahl procedure^40^. Lipid content was measured by Soxhlet extraction method using n-hexane as a solvent^41^. Ash content was determined based on the AOAC standard methods^42^.

### Statistical analysis

Three replications were performed for each experiment and the results were analyzed with SPSS software (SPSS, Inc., IBM, Somers, NY, USA, Version 22.0), (https://www.ibm.com/support/pages/downloading-ibm-spss-statistics-22). The statistical analysis of data was performed using one-way analysis of variance (ANOVA) and differences were analyzed by Duncan post-hoc test considering the significance at p $\le$ 0.05.

## Results

### In-silico identification, expression and purification of the novel PersiXyn8

**Figure 1:**
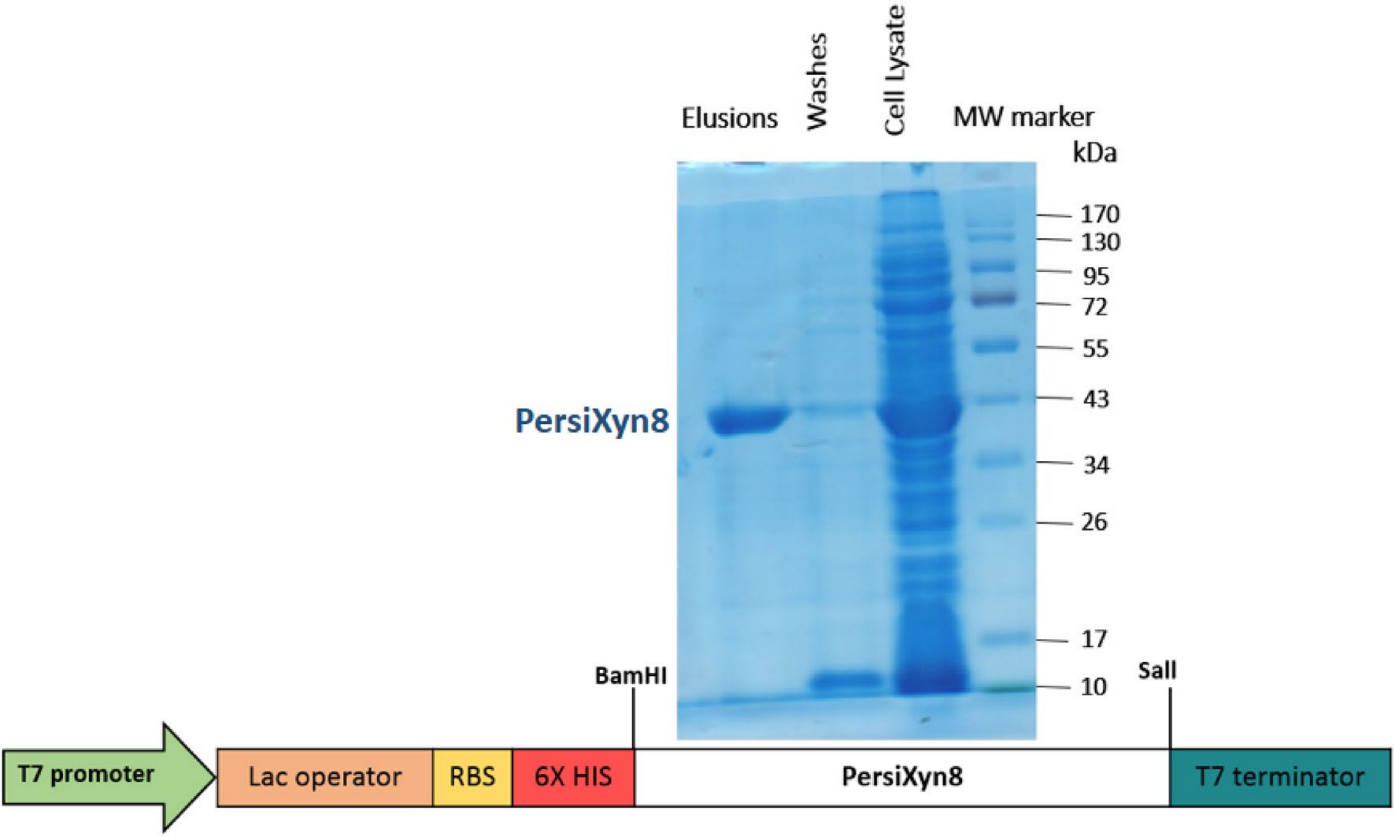
Schematic illustration map of the pET-28a ( +) expression vector used in this study and sodium dodecyl sulfate-PAGE (SDS-PAGE) of the purified PersiXyn8 is shown.

The metagenomic xylanase predicted by TAXyl was named PersiXyn8. The TAXyl predicted it as thermophile xylanase. The CDD predicts the PersiXyn8 as an endo-1,4-beta-xylanase from GH 10 family that has a high compatibility with the Pssm-ID 366032 with Bit Score 346.18 E-value 2.96e-119. Also, according to Phyre 2 server, an endo-beta-1,4-xylanase from *Cellvibrio japonicus* with 1US3 PDB code was the most similar structure to the PersiXyn8 with 100% confidence. The specific primers harboring *BamH*I and *Sal*I restriction enzymes were designed from cattle rumen metagenomic data to isolate PersiXyn8. The CDS of PersiXyn8 were amplified by Taq DNA polymerase in the BioRad thermocycler instrument. To increase the efficiency, accuracy, and, convenience two round cloning process were done. The first 1065 bp PCR fragment with 3´A overhang was gel purified and subsequently was cloned in pTZ57/RT. Second, the recombinant vector was digested with *BamH*I and *Sal*I restriction enzymes. Gel purified 1041 bp PersiXyn8 with *BamH*I and *Sal*I overhang was cloned in similarly digest purified pET-28a ( +) vector. For protein expression, *E. coli* BL21 (DE3) were transformed with recombinant pET-28a ( +) PersiXyn8 (Fig. 1). The small-scale expression showed that the induction at 25 °C, 18 h resulted in higher expression than that at 28 °C. Due to the higher expression, induction at 25 °C for 18 h was chosen for scaling up. The large scale (5*250 cc) expressed recombinant N-terminal His6-tagged PersiXyn8 was purified with the Ni–NTA Fast Start Kit and the purity of the protein was checked by the SDS-PAGE (Supplementary Fig. 2). The purified PersiXyn8 was beheld as judged by 12% sodium dodecyl sulfate- PAGE (SDS-PAGE), where a single band with a weight corresponding to the calculated 43 kDa (Fig. 1).

### Biochemical properties of the purified PersiXyn8

**Figure 2:**
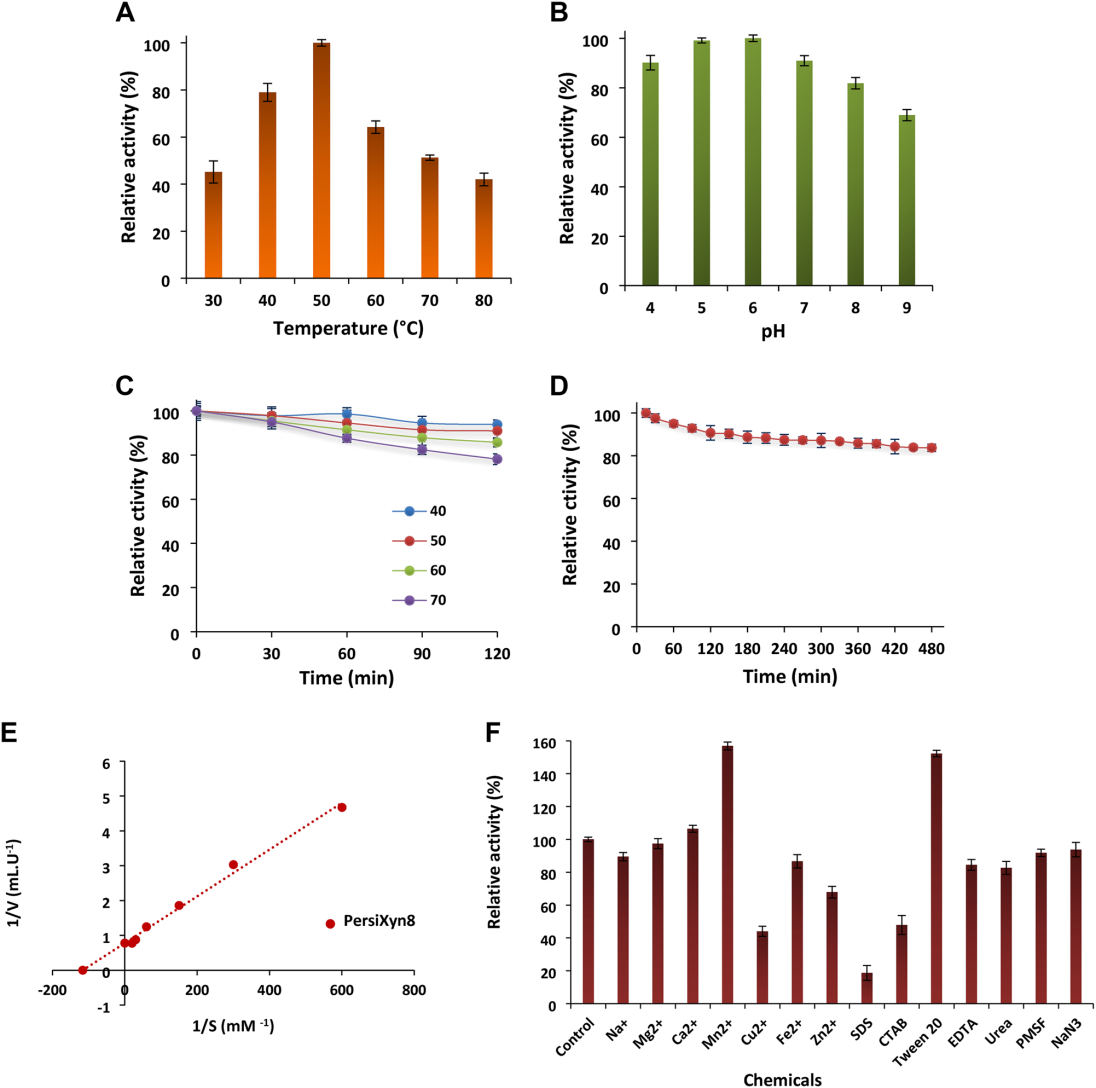
Biochemical characterization of the PersiXyn8. (**A**) Effect of temperature on the activity of the PersiXyn8 after incubation at 30 ℃–80 ℃ for 20 min at pH 6.0. (**B**) Effect of pH on the activity of the PersiXyn8 after incubation in pH 4.0–9.0 for 20 min at 50 ℃ and using the beechwood xylan as substrate. (**C**) Effect of temperature on the stability of the PersiXyn8 after 120 min incubation at temperature from 40 ℃ to 70 ℃. (**D**) Storage stability of the PersiXyn8 after 480 min incubation at 50 ℃. (**E**) Lineweaver–Burk plot of the purified thermostable PersiXyn8. (**F**) Effect of metal ions, surfactants and inhibitors on the activity of PersiXyn8 after 30 min pre-incubation of enzyme and additives at room temperature.

The purified xylanase was active over a wide range of temperatures and pH. As illustrated in Fig. 2A, the PersiXyn8 was active under temperatures 30 to 80 °C and exhibited the highest activity at 50 °C, which showed the optimum temperature for the enzyme activity. Moreover, the xylanase was active under high temperatures and could retain 42% of its maximum activity at 80 °C. The activity of xylanases in a broad temperature range is the major concern for effective hydrolysis of substrate and enzyme application in industry. Also, the optimal pH of the PersiXyn8 was found when it was incubated at pH 6.0 (Fig. 2B). The xylanase was active over a wide pH range (4.0–9.0) and remained its activity under both acidic and alkaline conditions, showing 90.15% and 68.93% activities at pH 4.0 and pH 9.0, respectively. Regarding the thermostability studies of the enzyme, it remained active and stable under high temperatures after 120 min of incubation. As shown in Fig. 2C, the PersiXyn8 showed 93.82% and 90.97% activities after 120 min incubation at 40 °C and 50 °C. Also, the enzyme remained most of its activity at higher temperatures showing 85.74% and 78.14% under 60 °C and 70 °C, respectively. These results confirm that the PersiXyn8 is thermostable xylanase.

Besides, the storage stability of xylanase was investigated by measuring the enzyme activity after 480 min incubation at 50 °C. According to the results from Fig. 2D, the PersiXyn8 was highly stable during 480 min of storage and showed 83.73% activity at the end of storage. The results of long-term stability in high temperature confirmed the capability of the PersiXyn8 for many applications which the most important are lignin-based and animal feed industries.

The kinetic studies showed that the enzyme obeys Michaelis–Menten kinetics when the beechwood xylan was used as substrate (Fig. 2E). Concerning the outcomes, the K_m_, K_cat_ and K_cat_/K_m_ of the purified xylanase calculated 0.0086 mM, 1717.72 s^−1^ and 199,640.15 1.mM^−1^.s^−1^, respectively.

The activity of the PersiXyn8 in the presence of metal ions and inhibitors was measured (Fig. 2F). The highest enzymatic activity was found in the presence of Mn^2+^ which stimulated the PersiXyn8 activity to 156.87%. Addition of the Ca^2+^ (106.47%), Mg^2+^ (97.45%), Na^+^ (89.47%) and Fe^2+^ (86.70%) didn’t show any observable change in the activity of xylanase. The Cu^2+^ inhibited the activity of PersiXyn8 to 44.04%. Among the surfactants and detergents, the maximum relative activity was obtained by the Tween 20 (152.25%). The activity of the PersiXyn8 was slightly decreased in the presence of urea (82.65%), PMSF (91.79%) and NaN_3_ (93.75%). The SDS and CTAB decreased the enzymatic activity to 18.72% and 47.86%, respectively. The enzyme maintained its activity when the chelating agent, EDTA was added and demonstrated the 84.50% relative activity.

### Substrate spectrum of the PersiXyn8

**Figure 3:**
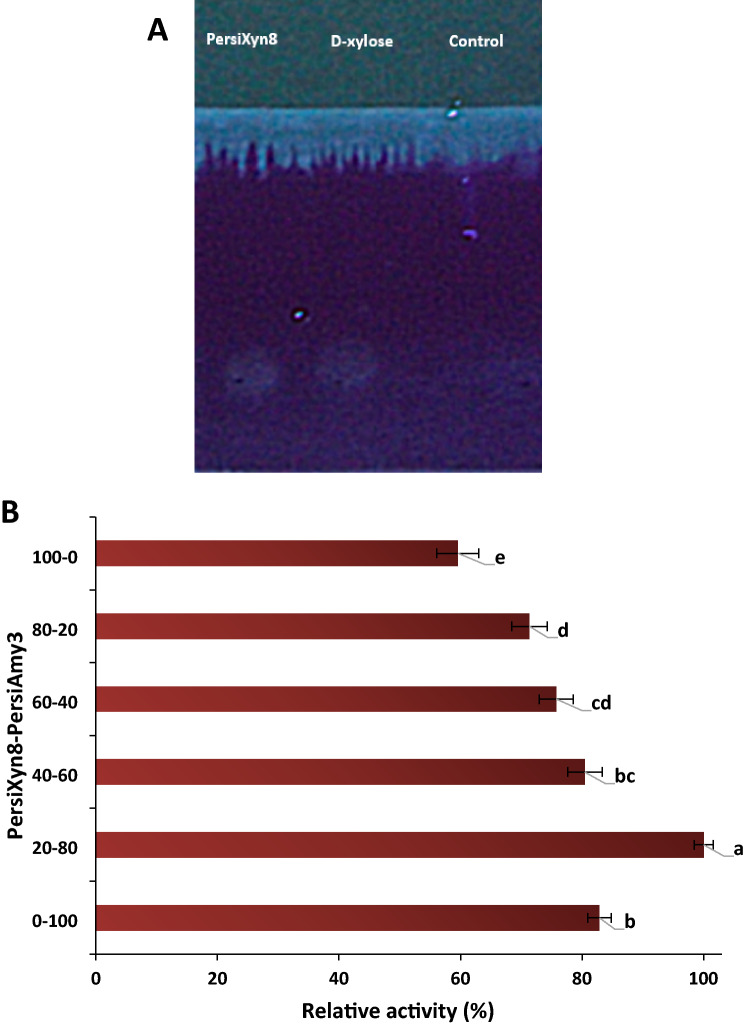
(**A**) Thin layer chromatography (TLC) analysis of hydrolysis products released from beech wood xylan by purified PersiXyn8, unhydrolyzed xylan was used as control. (**B**) Synergistic relationship between PersiXyn8 and PersiAmy3. Means with different letters are significantly different (p < 0.05).

Substrate specificity of the PersiXyn8 was determined using various substrates. Based on the results, the enzyme could hydrolyze all substrates, but low activity was detected in the presence of $\upbeta$-glucan (3506.76 U/mg), LBG (3937.76 U/mg) and CMC (3585.12 U/mg) (Table.1). The xylanase was most active in the presence of avicel (22,940.90 U/mg) followed by beechwood xylan (17,768.91 U/mg) and filter paper (17,239.96 U/mg). To ensure the hydrolysis of the beechwood xylan by the PersiXyn8, the TLC analysis was performed and showed the substrate backbone cleaved and xylooligosaccharide and small amounts of xylose were released (Fig. 3A).

**Table 1:** Specific activity of the PersiXyn8 on various substrates.

Substrate	Main linkage type	Specific activity (U/mg)
Avicel	(β-1,4) glucosidic	22,940.90
${\varvec{\upbeta}}$-glucan	(β-1,3) and (β-1,4) glucosidic	3506.76
Beechwood xylan	(β-1,4) xyloside	17,768.91
Filter paper	(β-1,4) glucoside	17,239.96
LBG	(β-1,4) glucoside	3937.76
CMC	(β-1,4) glucosidic	3585.12

### Synergistic relationship of the PersiXyn8 and PersiAmy3

In our previous study, the novel $\mathrm{\alpha }$-amylase (PersiAmy3) was used for biodegradation of the corn-based poultry feed which enhanced the hydrolysis of this material^26^. In order to increase the poultry feed conversion, the enzyme cocktail of PersiXyn8 and PersiAmy3 was prepared. Based on the results from Fig. 3B, simultaneous action and synergistic relationship were observed after combining these two enzymes in different ratios (p $\le$ 0.05). The highest synergistic effect of the enzyme cocktail was at a ratio of 80:20 (PersiXyn8:PersiAmy3). In other words, the highest activity at a ratio of 80:20 (PersiXyn8:PersiAmy3) showed the highest reducing sugars from poultry feed compared with the xylanase (60%) or $\mathrm{\alpha }$-amylase (83%) alone. This result showed the effectiveness of the enzyme cocktail for the degradation of the corn-based poultry feed.

### Patterns of sugar release comparison in degrading the poultry feed

**Figure 4:**
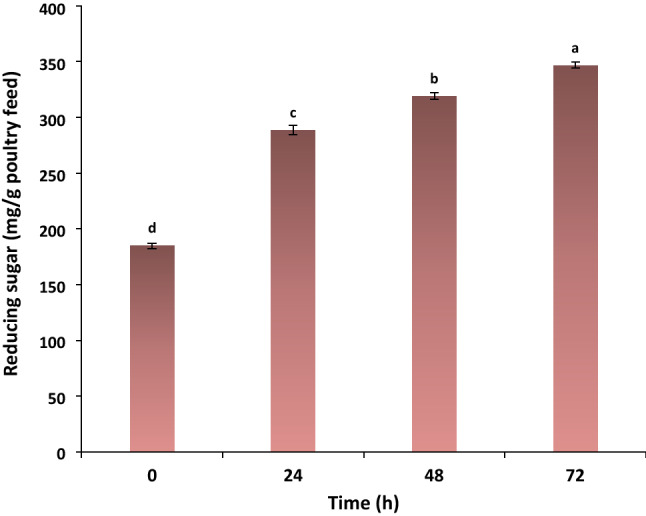
The amounts of reducing sugars during the poultry feed biodegradation with the enzyme cocktail after 72 h of hydrolysis at 50 °C.

The effect of the enzyme cocktail on poultry feed conversion was studied during 72 h of hydrolysis (Fig. 4). There was a continual increase in the release of reducing sugars during hydrolysis. The amount of 184.85 mg/g reducing sugars were generated before the incubation. This value increased to 288.54 mg/g and 319.13 mg/g after 24 h and 48 h of hydrolysis, respectively. At the final incubation time (72 h), reducing sugars reached 346.73 mg/g.

A significant increase in the amounts of released reducing sugars (p $\le$ 0.05) during the hydrolysis of poultry feed proved the effectiveness of combined PersiXyn8 and PersiAmy3 for SSF process.

### Surface morphology

In this study, the poultry feed is composed of 614 kg/ton of corn. This compound is the major and the most energy-yielding part of poultry feed (Supplementary Table.1). In this regard, the corn flour was selected for the SSF in the presence of a thermostable enzyme cocktail. Then, the effects of these operations on the antioxidant and nutritional properties of corn were studied.

**Figure 5:**
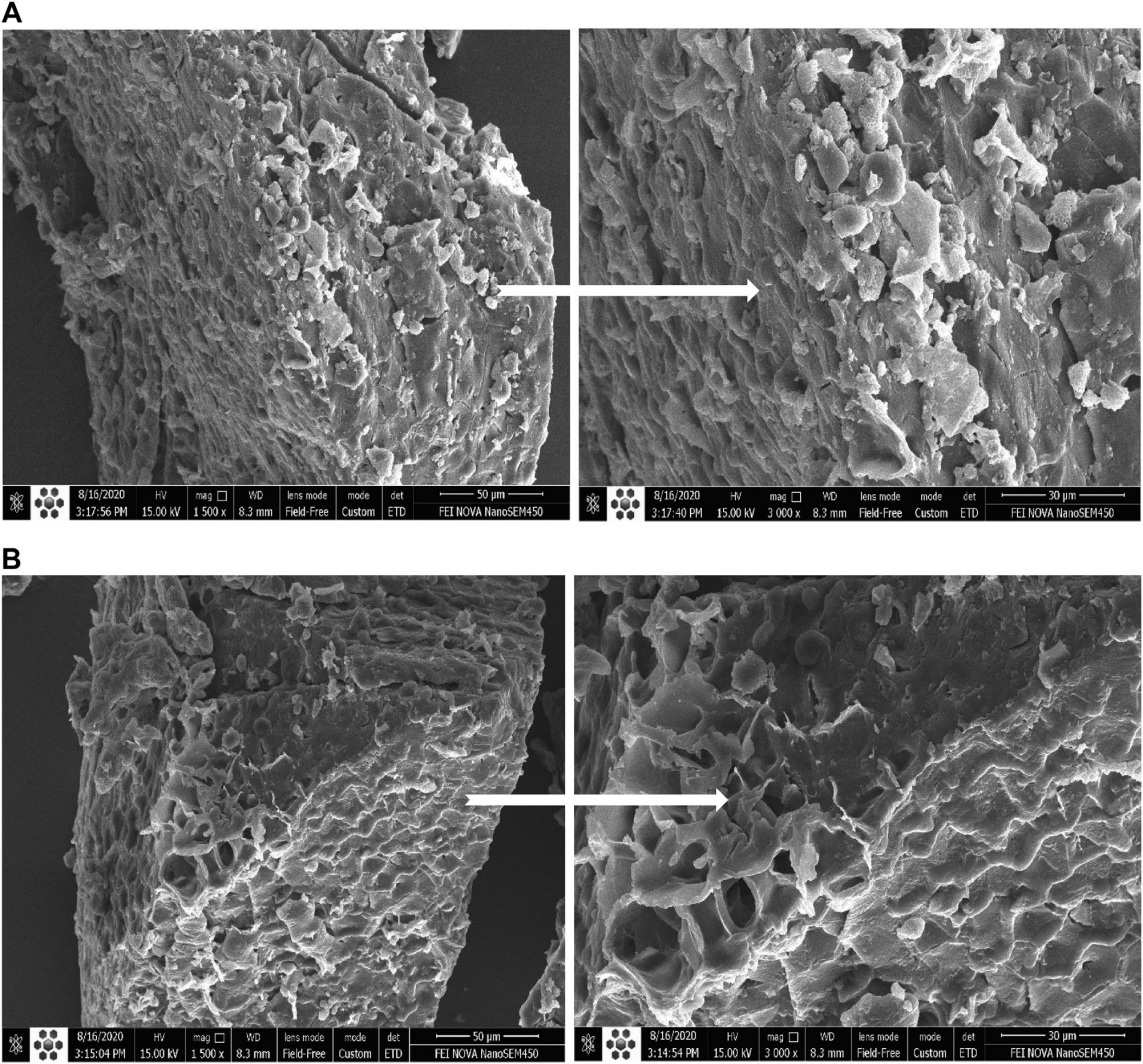
Scanning electron micrographs of unfermented corn (**A**) and corn after SSF and enzyme cocktail treatment (**B**). Left: under a magnification of 1500 $\times$. Right: under a magnification of 3000 $\times$.

For investigating the structural changes during fermentation and enzyme treatment, the corn flour before and after fermentation in the presence of enzymes was subjected to SEM study (Fig. 5). The unfermented sample (control) showed compact spherical, intact granules with convex surfaces (Fig. 5A). The addition of the enzyme cocktail in the fermentation broth changed the structure of the substrate. As is apparent in Fig. 5B, the native arrangement of the corn was destroyed and large holes and porous blocks were produced. It indicated that the surface structure of corn was damaged during the fermentation and α-amylase and xylanase activities. These processes lead to the release of phenolics and increase the nutritional and bioactive properties^43^.

### Phenolic content and antioxidant activity of the fermented samples

**Figure 6:**
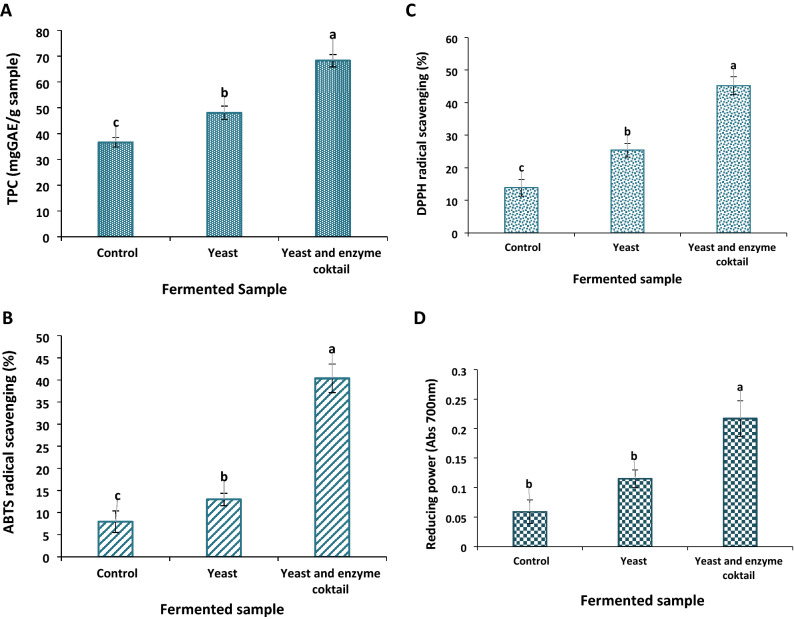
Phenolic content and anti-oxidant activities of the control, yeast added and fermented-enzyme cocktail treated samples. (**A**) Total phenol content. (**B**) Radical scavenging rate by ABTS. (**C**) Radical scavenging rate by DPPH. (**D**) Reducing power. Means with different letters are significantly different (p < 0.05).

The total phenol content of the solid-state fermented samples was changed significantly (p $\le$ 0.05). As presented in Fig. 6A, the uninoculated sample showed 36.60 mg GAE/g total phenol that rose to 48.04 mg GAE/g in the presence of yeast. The addition of the enzyme cocktail improved the hydrolysis of corn and enhanced this amount to 68.23 mg GAE/g. The phenolic compounds are bound to proteins or polysaccharides in the substrate. The activity of the combined PersiXyn8 and PersiAmy3 was beneficial for facilitating the release of phenolic compounds during SSF.

The ABTS and DPPH free radical scavenging activities and reducing power of samples were determined. The enzyme-treated sample showed 40.36% and 45.21% ABTS and DPPH radical scavenging activities after fermentation. The control sample showed 7.91% and 13.82% ABTS and DPPH radical scavenging, which increased to 12.95% and 25.39% due to yeast action (Fig. 6B,C). Similar improvement was observed in reducing power of samples. The absorbance was gradually increased from 0.059 in the control sample to 0.217 (Abs700 nm) in the presence of enzyme cocktail and yeast (Fig. 6D). These results confirmed the potential of carbohydrate-hydrolyzing enzyme cocktail in improving the bioactive properties of fermented corn for poultry feed supplementation.

### Protein, lipid and ash contents of the fermented samples

The current study investigated the essential nutritional parameters of fermented corn including protein, lipid and ash contents (Table 2). The presence of PersiXyn8 and PersiAmy3 significantly increased the nutritional values of fermented corn. Protein is a critical factor in the feed that enhanced from 7.18% in the control sample to 7.75% in the fermented sample. After the addition of the enzyme cocktail, this value improved to 12.06%. This result showed that the SSF process and effect of the enzyme cocktail increased the protein content by 4.88%. The activity of the yeast could explain this during the metabolization of the substrates and the increase in the release of fermentable sugars by enzymatic activity. Moreover, the activity of the enzyme cocktail during fermentation was also increased the lipid content from 3.54% to 4.87%. The ash content represented 2.36% in the control sample. The yeast activity under SSF increased this amount to 7.68% which improved to 8.82% due to enzymatic activity.

**Table 2:** Protein, lipid and ash contents of the fermented samples. Different letters in the same column indicate significant differences (p < 0.05).

	Protein (%)	Lipid (%)	Ash (%)
Control	7.18 $\pm$ 0.02^c^	3.54 $\pm$ 0.08^c^	2.36 $\pm$ 0.07^c^
Fermented	7.75 $\pm$ 0.03^b^	4.70 $\pm$ 0.11^b^	7.68 $\pm$ 0.12^b^
Fermented + enzyme cocktail	12.06 $\pm$ 0.02^a^	4.87 $\pm$ 0.07^a^	8.82 $\pm$ 0.07^a^

## Discussion

One of the important goals in the poultry industry is the feed efficiency of the birds. The carbohydrate-hydrolyzing enzymes are used to reduce the cost of feeding and make the nutrients available for poultry utilization^44^. For this purpose, this study identified a thermostable PersiXyn8 from rumen metagenomic data. The biochemical properties of the novel xylanase suggest that it is a good candidate for industrial application. The high activity of the PersiXyn8 over a broad range of temperature and pH was confirmed when compared with the activity of xylanases in the literature. The PersiXyn8 retained 42% of its activity at 80 °C while xylanase from Holstein cattle rumen metagenomic library showed < 10% activity at 80 °C^45^. The PersiXyn8 also showed high activity over a wide range of pH (90.15% at pH 4.0 and 68.93% at pH 9.0) while the xylanase from the metagenomic DNA of cattle dung compost showed 40% activity at pH 4.0 and < 20% activity at pH 9.0^46^. As compared to the xylanase isolated by the culture-dependent method, xylanase from *A. kamchatkensis* exhibited < 20% activity at pH 4.0 and temperature of 80 °C^34^. These results showed the potential of PersiXyn8 to be used in the food industry and supplementation of feeds^47,48^. The stability of PersiXyn8 remained considerably high when incubated for quite a long period under high temperatures compared to the previous studies. Xylanase from an extreme temperature hot spring metagenome showed less than 70% activity after 5 h incubation at 50 °C^49^, while the PersiXyn8 showed 87.16% activity after 5 h at this temperature. Another novel thermostable xylanase from *Chaetomium* showed less than 70% activity after 30 min incubation under 70 °C^50^. In another study, a thermostable and alkaline tolerant xylanase from *Bacillus* was completely inactivated after 10 min incubation at 70 °C^51^. Comparing the thermal stability of the PersiXyn8 with previously reported xylanases proved another important property of the enzyme for industrial purposes.

The kinetic studies showed the low value of K_m_ for the PersiXyn8 which revealed the high affinity of the enzyme towards the beechwood xylan. This result is lower than a previous study which found K_m_ of 0.248 mM for the novel xylanase from fungi^52^. The K_cat_ and K_cat_/K_m_ values of the PersiXyn8 were higher than the results reported for xylanase from gut metagenome using beechwood xylan as substrate^53^. The PersiXyn8 was also active in the presence of various metal ions and inhibitors increasing the number of applications for the enzyme^49,54^. Another ability of the PersiXyn8 was its wide substrate range which is highly demanding for industrial purposes. Specificity studies indicate that the xylanase has the highest specificity activity to beechwood xylan and avicel. This result was in agreement with xylanase from an extreme temperature hot spring metagenome^49^ and xylanases from *Fusarium*^55^ which showed. TLC analysis of enzymatic hydrolysis products indicated that the PersiXyn8 degraded the beechwood xylan and liberate xylooligosaccharide and small amounts of xylose. These observations confirmed the capability of the PersiXyn8 to be used for hydrolysis of lignocellulosic materials.

Production of the enzyme cocktail is an efficient technique to increase the enzymatic activity and effective hydrolysis of substrate^19^. In our previous study, the novel $\mathrm{\alpha }$-amylase (PersiAmy3) was used for biodegradation of the corn-based poultry feed which enhanced the hydrolysis of this material^26^. To increase the PersiXyn8 activity, it was combined with the PersiAmy3, which improved the enzymatic hydrolysis efficiency of poultry feed. Researchers have been studied the effect of xylanase on the hydrolysis of poultry feed. For instance, Alokika et al. found 95.54 mg/g substrate reducing sugar after 48 h of poultry feed hydrolysis using 10 U/g xylanase and Panwar et al. observed the maximum amounts of 81 mg/g reducing sugar after 1 h of the poultry feed hydrolysis with 200 U/g xylanase^56,57^. Other studies investigate the effect of multienzyme preparations in improving the poultry feed. Supplementation of poultry feed with amylase and xylanase increased the starch digestibility and growth performance in broilers^58,59^. Enzyme supplementation has both environmental and economic benefits for poultry producers. Xylanase hydrolyzes hemicellulose and allows for better utilization of dietary energy^60^. On the other hand, α-amylase had a positive effect on feed conversion^59^. These enzymes increase the release of trapped nutrients, increasing energy utilization and reducing the cost of production^61^. Therefore, the production of the enzyme cocktail containing xylanase and α-amylase is advantageous for improving the poultry industry.

Corn is one of the most common feedstuffs and source of carbohydrate and protein^62^. Thus, improving the nutritional value of this substrate is considerable. This seed contains approximately 9% of non-starch polysaccharide^63^. Degradation of the non-starch polysaccharide by carbohydrate-hydrolyzing enzymes during solid-state fermentation improves digestibility and the nutrient availability of poultry feed. SEM micrographs of corn seeds showed the structural changes during fermentation in the presence of a carbohydrate-hydrolyzing enzyme cocktail. The major phenolic compounds of the plant material are bound to cell wall structures such as cellulose and hemicellulose^64^. It has been reported that the activity of carbohydrate-hydrolyzing enzymes such as amylase and xylanase is connected to the liberation of phenolic compounds^4^. In this study, the enzyme addition influenced the total phenolic content of corn seeds and improved the antioxidant activity due to the increase in the availability of phenolic compounds. Presence of the polyphenols in the animal diet increases the antioxidant activity, animal performance, and/or meat sensory properties^65^. In a previous study, SSF of the corn showed a linear correlation between total phenol content, DPPH and ABTS scavenging activities^66^. Moreover, the positive effect of the SSF as an environmentally friendly and cost-beneficial process in increasing the antioxidant activity of phenolic compounds was stated^67^.

Improving the nutritional value of poultry feed have versatile and important effects on the health and growth of animals. It has been reported that the protein content of corn showed 7.13–8.21% increase after 30 days SSF by *A. brasiliensis* and *A. bisporus*^68^. The previous study mentioned the increase of ash content from 3.77 to 4.71% and total protein from 0.49 to 0.55% during the SSF of the corn-based poultry feed^69^. In another report, SSF of the soybean/corn feed showed 1.3−4.2% higher protein contents compared with control^12^. Our results showed a significant increase in protein and ash contents of the corn powder as a result of fermentation and enzyme treatment. Concerning these outcomes, it is evident that the SSF of corn in the presence of the PersiXyn8 and PersiAmy3 is a beneficial way of improving the nutritional quality for further supplementation of the corn-based poultry feed.

## Conclusion

Carbohydrate- hydrolyzing enzymes can increase the hydrolysis of cellulose and hemicellulose and be used to produce high-value products. The development of synergistic enzyme cocktail using the thermostable xylanase and $\mathrm{\alpha }$-amylase is a practical way to increase the efficiency of enzyme and improve their potential in the poultry industry. In this work, the thermostable carbohydrate-hydrolyzing enzyme cocktail was prepared using the novel PersiXyn8 and PersiAmy3, identified from metagenomic data. The enzyme cocktail was effectively used for the hydrolysis of poultry feed. During the SSF process in the presence of a thermostable enzyme cocktail the substrate was disrupted which was observed in the SEM analysis. The highest phenolic content was observed in the fermented and enzyme treated sample. The antioxidant capacity was increased remarkably in the presence of combined PersiXyn8 and PersiAmy3 due to the higher reducing power and free radical scavenging capability. Protein, lipid, and ash contents of the fermented corn were increased and confirmed the improved nutritional value. Outcomes revealed the potential of the carbohydrate-hydrolyzing enzyme cocktail under SSF for improving the bioactive and nutritional properties of corn to be used as a supplement in corn-based poultry feed.

## Supplementary Information

Supplementary Information 1.

Supplementary Information 2.

## Data Availability

Sequence data of this study have been deposited in the GenBank accession number of MW349589.

## Abbreviations

SSF: Solid-state fermentation
ABTS: 2,2'-Azino-bis (3-ethylbenzothiazoline-6-sulfonic acid
DPPH: 2,2-Diphenyl-1-picrylhydrazyl
DNS: 3,5-Dinitrosalicylic acid
BSA: Bovine serum albumin
LBG: Locust bean gum
CMC: Carboxymethylcellulose
EDTA: Ethylenediaminetetra acetic acid
PMSF: Phenylmethylsulfonyl fluoride
SDS: Sodium dodecyl sulfate
CTAB: Cetrimonium bromide
SEM: Scanning electron microscope
TAXyl: Thermal activity prediction for xylanase
SDS-PAGE: Sodium dodecyl sulfate polyacrylamide gel electrophoresis
