# Author Correction: In vitro bioprocessing of corn as poultry feed additive by the influence of carbohydrate hydrolyzing metagenome derived enzyme cocktail

**DOI:** 10.1038/s41598-022-05555-7

**Published:** 2022-01-17

**Authors:** Seyed Hossein Mousavi, Seyedeh Fatemeh Sadeghian Motahar, Maryam Salami, Kaveh Kavousi, Atefeh Sheykh Abdollahzadeh Mamaghani, Shohreh Ariaeenejad, Ghasem Hosseini Salekdeh

**Affiliations:** 1 Department of Systems and Synthetic Biology, Agricultural Biotechnology Research Institute of Iran (ABRII), Agricultural Research Education and Extension Organization (AREEO), P. O. Box, 31535-1897 Karaj, Iran; 2 Department of Food Science and Engineering, University College of Agriculture & Natural Resources, University of Tehran, Karaj, Iran; 3 Laboratory of Complex Biological Systems and Bioinformatics (CBB), Department of Bioinformatics, Institute of Biochemistry and Biophysics (IBB), University of Tehran, Tehran, Iran; 4 Departmen of Molecular Sciences, Macquarie University, Sydney, NSW Australia

Correction to: *Scientific Reports* 10.1038/s41598-021-04103-z, published online 10 January 2022

The original version of this Article contained an error in the spelling of the author Seyedeh Fatemeh Sadeghian Motahar, which was incorrectly given as Seyedeh Sadeghian Sadeghian Motahar.

The Author contributions statement now reads:

“S.H.M., S.F.S.M. and A.S.A.M. carried out the experimental procedures. M.S. contributed in data analysis and interpretation. K.K. performed bioinformatic analysis. S.A. and G.H.S. designed the study and helped with data interpretation and discussion. All the authors wrote and reviewed the article.”

The original Article has been corrected.

