# Supplementary material for: In vitro bioprocessing of corn as poultry feed additive by the influence of carbohydrate hydrolyzing metagenome derived enzyme cocktail
Source: Sci Rep. 2022 Jan 10;12:405. doi: 10.1038/s41598-021-04103-z (PMC8749004; doi:10.1038/s41598-021-04103-z)

**Supplementary material**

**In vitro bioprocessing of corn as poultry feed additive by** **the influence of carbohydrate hydrolyzing metagenome derived enzyme cocktail**

Seyed Hossein Mousavi^a^, Seyedeh Fatemeh Sadeghian Motahar^b^, Maryam Salami^b^, Kaveh Kavousi^c^, Atefeh Sheykh Abdollahzadeh Mamaghani^a^, Shohreh Ariaeenejad^a^*and Ghasem Hosseini Salekdeh^a,d^*

**^a^** Department of Systems and Synthetic Biology, Agricultural Biotechnology Research Institute of Iran (ABRII), Agricultural Research Education and Extension Organization (AREEO), Karaj, Iran.

^b^ Department of Food Science and Engineering, University College of Agriculture & Natural Resources, University of Tehran, Karaj, Iran

**^c^** Laboratory of Complex Biological Systems and Bioinformatics (CBB), Department of Bioinformatics, Institute of Biochemistry and Biophysics (IBB), University of Tehran, Tehran, Iran.

**^d^** Department of Molecular Sciences, Macquarie University, Sydney, NSW Australia.

* Corresponding authors:

Shohreh Ariaeenejad (shariaee@gmail.com; sh.ariaee@abrii.ac.ir)

And

Ghasem Hosseini Salekdeh ([h_salekdeh@abrii.ac.ir](mailto:h_salekdeh@abrii.ac.ir); hsalekdeh@yahoo.com )

Department of Systems and Synthetic Biology, Agricultural Biotechnology Research Institute of Iran (ABRII), Karaj, Iran.

P. O. Box: 31535-1897, Tel.: +98 26 32703536, Fax: +98 26 32704539.

Supplementary Table.1. Poultry feed ingredient and nutrient composition.

A) Feed ingredients per ton of grain.

B) Nutrient compositions of diets which determined in the lab based on AOAC methods, and metabolizable energy determined by calculation based on the results.

| A |  | |
| --- | --- | --- |
| Ingredients | **kg in 1ton grain** | |
| Corn | 614 | |
| Soy | 340 | |
| Oil | 10 | |
| Calcium carbonate | 6 | |
| Consternate | 30 | |
| Total | 1000 | |
| B |  | |
| Nutrient composition | **Lab** | **Real** |
| Energy (kcal/kg) | 0.91 | 2.7 |
| Protein (%) | 15 | 18 |
| Calcium (%) | 7.7 | 13 |
| Total phosphorus (%) | 6.58 | 11.7 |
| Available phosphorus (%) | 5.86 | 11.05 |
| Sodium (%) | 5.19 | 5.19 |
| Choline (mg/kg) | 10 | 10 |
| Lysin (%) | 4.78 | 5.26 |
| Methionine (%) | 12.11 | 12.15 |
| Cystine (%) | 0.02 | 0.14 |
| Methionine+ Cystine (%) | 12.13 | 12.30 |
| Threonine (%) | 0.07 | 0.59 |
| Tryptophan (%) | 0.024 | 0.15 |

Supplementary Fig.1: Amino acid alignment between PersiXyn8, MW349589 (QYR68957.1) and ten xylanases deposited in NCBI with highest similarity scores. The first item is PersiXyn8 itself.

CLUSTAL 2.1 multiple sequence alignment

WP_035668525.1 ----MNQQLNTPDLYQVYKNFFTIGAAVNSKTLESEKELLKKHYNSLTAENEMKFELLQP

WP_096187652.1 MVVTNSVSHSIPSLCDVYKDYFLIGAAVNQQTLQTEQNLIKKHFNSLTAENEMKFEHLQP

WP_158322277.1 --MSEENYNEVLSLCERYREQFFIGAAVNQYTIDTQQELLKKHFNSITAENEMKFENLQP

WP_183255100.1 ------MQERLPSLCETYKDYFHIGAAVNPITIETQKQLLIDHVNSITAENHMKFEHLQP

WP_213519084.1 -------MNQVPKLWEVYKDYFDIGAAVNLNTIKSSEQLLQTHFNSITAENDMKFVSVHP

WP_201006369.1 ----MSSISKLLALRSVYKDYFDIGAAVNLTTIESQKELLTAHYNSVTAENDMKFESVHP

NLH13424.1 ----MSSDQQVVSLCDAYKDYFPIGAAVSSRCIHTHRDLLLRHFCSVTATNEMKFSSIHP

NLU41745.1 ------MAGEVVSLCKAYERFFPIGSAVSARSIRTHRDLLLKHFSSITPSNEMKFSSVHP

WP_188880953.1 ----MNQDGNIPSLAAAYAAYFPIGAAVSPEVIRSHRDVLTRHFSSVTAENHMKFALVHP

WP_198543709.1 ----------MQSLWEAYKDFFPIGAAVNAETVKTHEELLIKHFNSLTAENEMKFSSLHP

PersiXyn8 ---MNEWEKELMGLCEIYKDYFPIGAAVSARRIKRYESLLKKHFNSVTPENALKMINVYK

QYR68957.1 ---MNEWEKELMGLCEIYKDYFPIGAAVSARRIKRYESLLKKHFNSVTPENALKMINVYK

* * * **:**. : ..:: * *:*. * :*: :

WP_035668525.1 EQGNFNFANADKLVAFANENNMKLRGHTLVWHNQTTEWLFQNPDGTQVNREILLQRMEEH

WP_096187652.1 AEGQFSFEYADKLVSFANDNGKKVRGHTLVWHNQTSDWLFTDSAGKTIDRETLLARMENH

WP_158322277.1 EEGKFTFAVADKLVSFAKENHKQVRGHTLVWHNQTPDWVFTNKNGSFADRKTVLERMNTH

WP_183255100.1 EEGEFTFETADQIVDFAVSNNMAVRGHTLVWHNQTPDWVFHDGQGRFVSREVLLERMKAH

WP_213519084.1 EEQRYTFEAADALMDFATANGKKMRGHTLVWHNQTTEWVFEGEHGQPATREQLLARMKAH

WP_201006369.1 SEEAYTFEAADKIADFAAANGMKLRGHTLVWHNQTPDWVFQNANGAPVDRETLLARMKSH

NLH13424.1 DEGSYQFVLADSIVEFAEANDMLIRGHTLVWHQQTPERVFIDSDGASASRDLLLSRMRDH

NLU41745.1 TESAFQFLFSDSIADFAQANGMLLRGHVLVWHQQTPQWVFEQADGSPASRDLLLDRMREH

WP_188880953.1 EPDRYDFAAADDIVAFAQANHMKVRGHTLVWHNQTPSWVFEGSDGSPVKPADLRARMREH

WP_198543709.1 QDGVYEFKDADLIHDFAKRHNMKLRGHTFVWHNQTPEWVFKNEDGTSVSRDLLLERLKAH

PersiXyn8 GPDKYNFEEMDQIVRFANDNNMLLRGHVLIWYSQVAESFFENDNGEMVSREMLLTRMKEY

QYR68957.1 GPDKYNFEEMDQIVRFANDNNMLLRGHVLIWYSQVAESFFENDNGEMVSREMLLTRMKEY

: * * : ** : :***.::*:.*... .* * : *:. :

WP_035668525.1 ISTVLGRYKGQFYSWDVVNEAISDDDSEYLRKSKWLDIIGEDFIAKAFEFAHQADPNASL

WP_096187652.1 IGTVVRRYKEEVYCWDVVNEAVADHSQALLRDSNWLSIIGEDFIEKAFHFAHSANPEALL

WP_158322277.1 ISEVVKRYKDDLYCWDVVNEVVTDEGPKLLRESKWLETIGEDFIDKAFEFAHQVDPDALL

WP_183255100.1 ISTVVQRYKGKVYCWDAVNEAVDDEGDDLLRPSKWRQIIGDDFIEQAFLYAHEADPDALL

WP_213519084.1 IDTVVGRYKGLIYAWDVVNEVIEDKSDVWLRDSKWLQLVGEDFIAKAFQYAHDADPNALL

WP_201006369.1 IDTVVGRYKGIVYCWDVVNEVIEDKSGVWLRESKWLNLAGEDFIAKAFEYAHAADPKALL

NLH13424.1 IETVVSRYKGRVYCWDVVNEAIDDKGSDYLRPSKWLQIIGPDYIEKAFEYAHAVDPDALL

NLU41745.1 IHTLVSRYRGKAYCWDVLNEAVDDKGTDYLRRTKWLEIIGPDYIEKAFQYAHDADPDALL

WP_188880953.1 IHTVMSRYRGRVYCWDVVNEAVADSGEAWLRDSKWHAILGPDYLRDAYIFAHEADPDALL

WP_198543709.1 VHDVVKRYGNDVYCWDVVNEAVEDKTAQYLRDTKWLEIIGESYIQKAFEIVNQAAPEAML

PersiXyn8 IRKVVGRYKKDIYCWDVVNEAIDDNDNIYLRDTQWKKIIGDDYIEQAFLIAHEMAPEASL

QYR68957.1 IRKVVGRYKKDIYCWDVVNEAIDDNDNIYLRDTQWKKIIGDDYIEQAFLIAHEMAPEASL

: :: ** *.**.:**.: * ** ::* * .:: .*: .: *.* *

WP_035668525.1 FYNDYNESHPNKRERIYRLVKSLLDKDVPIHGVGLQAHWNVHDPSLDDIRAAIERYASLG

WP_096187652.1 FYNDYNESDPDKSDKIFQLVCSLVEREVPIHGVGLQAHWNLYKPSYDEIRKAIEKYASLG

WP_158322277.1 FYNDYNESDPTKRDKIYTLVKGLKERGVPIHGVGLQAHWNVSSPSYDDIRRAIEKYATLE

WP_183255100.1 FYNDYNESFPEKREKIYKLVKSLRDKDIPIHGIGMQAHWNLTRPSLDEIRMAIERYASLG

WP_213519084.1 FYNDYNECNPVKRDKIIKLVQSLQEQDVPIHGIGLQAHWNLSGPSIAEIREAIEKYAALG

WP_201006369.1 FYNDYNECNPEKRDKIIRLVQSLQAKQVPIHGIGLQGHWNLNGPSLAEIREAIERYAATG

NLH13424.1 FYNDYNAVAPYKREKIMRLITELQQKGIPIHGIGLQGHWNIYWPSVDEIRYALDDYARLG

NLU41745.1 FYNDYNEVAPEKRKKIIRLVTELRAKDVPIHGIGLQGHWSIHSPSIDAIDEALEEYASLG

WP_188880953.1 FYNDYNECEEEKREKIYRLVRDLRAQDVPVHGIGMQGHWGLVRPAADDIRRTIERFASLG

WP_198543709.1 FYNDYNECVPEKREKIYKLVRTLKENGVPIHGIGLQCHWNIFNPSFDEIQRSLEKYASLG

PersiXyn8 FYNDYNYVIKNKREKIYKLIKSLKGRDIPIHGIGIQGHWGLYYPDVTEIKRTLELFADLD

QYR68957.1 FYNDYNYVIKNKREKIYKLIKSLKGRDIPIHGIGIQGHWGLYYPDVTEIKRTLELFADLD

****** * .:* *: * . :*:**:*:* **.: * * ::: :*

WP_035668525.1 VQLQITEMDVSMFSWDDRRADLTQPTEEMLHLQAERYDQFFRVFREYKEVISNVTFWGAN

WP_096187652.1 LQLHITEMDVSAFEWNDRRTDLKEPLKEMLYLQAERYDQFFQIFREYKDHITSVTFWGVS

WP_158322277.1 LQIQLTEMDVSVFEFGDKRKDLKKPTEEMLGAQAERYEEFFNIFREYSDVITGVTFWGAA

WP_183255100.1 VQLHITELDISMFEFNDHRKDLTNPTELMIEQQADRYGQIFSLFKEYRDVIQSVTFWGVA

WP_213519084.1 LQLQITELDVSVFNYEDRRTDLTAPTAQMLELQAERYAQIFELFREYKDVITAVTFWGAA

WP_201006369.1 LKLQVTELDISVFDHDDKRTDLTEPTADLLERQAERYGQVFELFREYKEAITAVTFWGAA

NLH13424.1 LTIQITELDLSVFAYGDTRIDLTEPTSEMMELQAKRYEELFRLFREYKGVISGVTFWGIA

NLU41745.1 LPLQITELDISVFSFADLRTDLMAPTDEMMKLQAEKYEKVFELFRRYRDVITGVTLWGTA

WP_188880953.1 VQIQITELDITLYDWQDRRTDLRTPPPEWLERQAERYGEIFAIFREYKDVITGVTLWGVA

WP_198543709.1 LKIQITEMDISVFTFEDQRRDLTEPTAEMIEKQASLYAKAFKLFREYKDVINGVTLWGVA

PersiXyn8 LKIQLTEMDMSMFEWGDNRRDMKQPSGEMLKIQAEKYGKIFEILREYAEVISGVTFWGVA

QYR68957.1 LKIQLTEMDMSMFEWGDNRRDMKQPSGEMLKIQAEKYGKIFEILREYAEVISGVTFWGVA

: :::**:*:: : * * *: * : **. * : * :::.* * **:**

WP_035668525.1 DSYTWLNDFPVRGRKNWPFVFGENGVPKESFWRIAKF---------

WP_096187652.1 DHYTWLNDFPVKGRRNWPFVLDHNGNPKPAFQRVCQVPGTSRIMSN

WP_158322277.1 DDYTWLSDFPVKGRKNWPLLFDEQHQPKQSFWRIMEF---------

WP_183255100.1 DDYTWLDNFPVKGRKNWPFLFDELHRPKPSFWRVHTC---------

WP_213519084.1 ENYTWLDDFPVRGRKNWPFVFDEQQQPKQSFWRIVDFAK-------

WP_201006369.1 DDYTWLDNFPVHGRKNWPFVFDAKHEPKASFGKITDWQK-------

NLH13424.1 DDVTWLHNFPVRGRRDWPLLFDCEHQPKESFYRVARF---------

NLU41745.1 DDHSWRHNFPVRGRRDWPLLFDFDHQPKESFFRVVNF---------

WP_188880953.1 DDWTWRDDFPVRGRKDWPLLFDTAHQPKAAFWRALP----------

WP_198543709.1 DDKTWLDDFPVRGRKNWPLLFDVEHNPKEAFYRIVQF---------

PersiXyn8 DDYTWLDDFPVEGRKDWPLLFDSELKPKPSLDAITRFSSIR-----

QYR68957.1 DDYTWLDDFPVEGRKDWPLLFDSELKPKPSLDAITRFSSIR-----

: :* :***.**::**:::. ** ::

Supplementary Fig.2: SDS-PAGE profile of the purified PersiXyn8 using 12 % polyacrylamide gels in attached file.


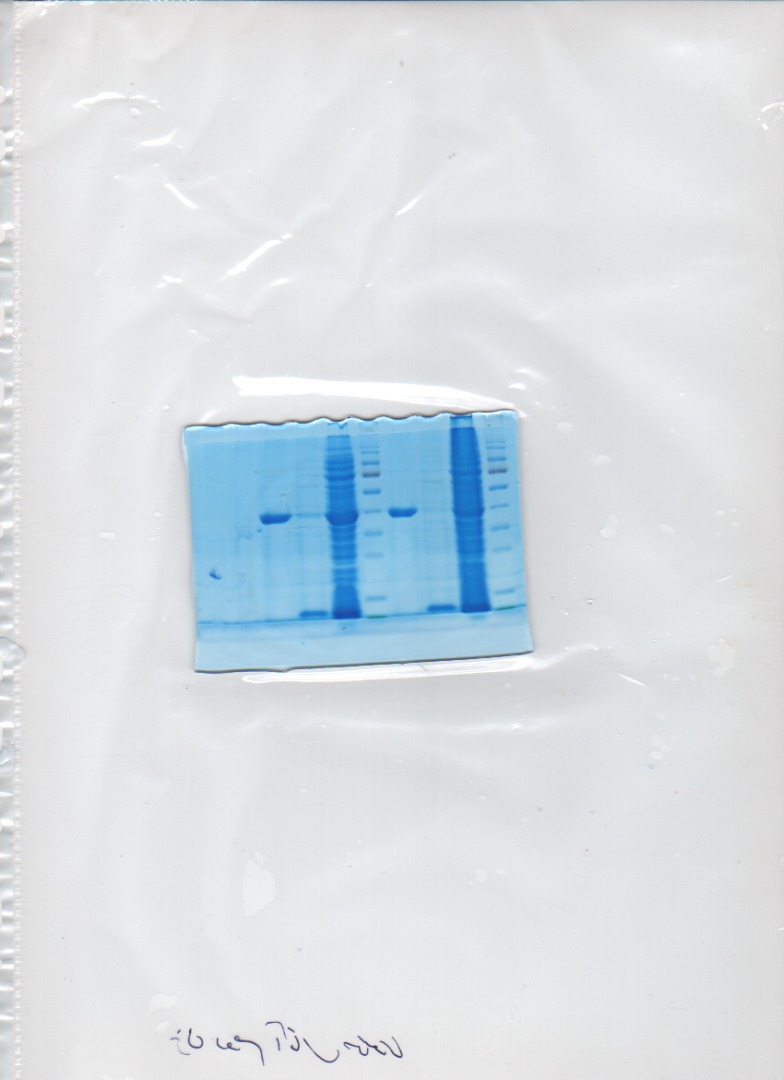

Supplement: Supplementary file 1 — Supplementary Information 1. [file 41598_2021_4103_MOESM1_ESM.docx]
