# Supplementary figures and images for: In vitro bioprocessing of corn as poultry feed additive by the influence of carbohydrate hydrolyzing metagenome derived enzyme cocktail
Source: Sci Rep. 2022 Jan 10;12:405. doi: 10.1038/s41598-021-04103-z (PMC8749004; doi:10.1038/s41598-021-04103-z)

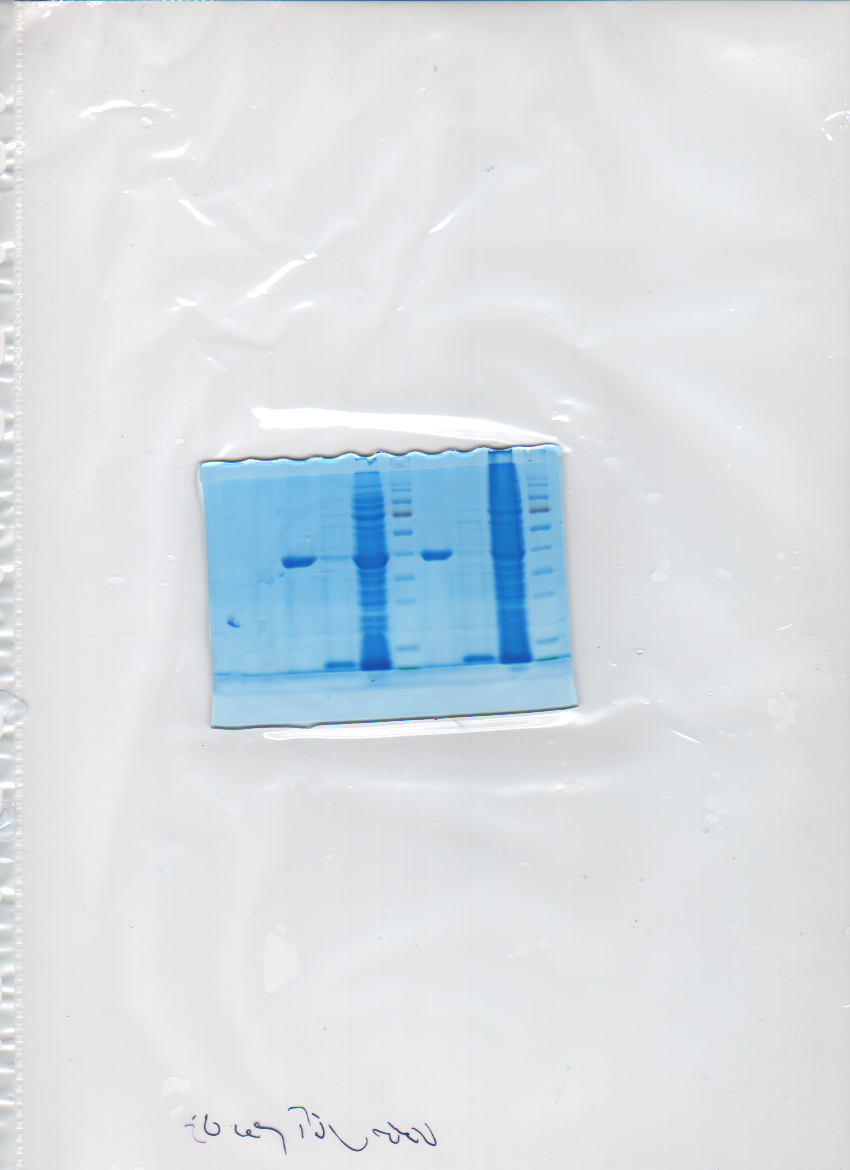

Supplement: Supplementary file 2 — Supplementary Information 2. [file 41598_2021_4103_MOESM2_ESM.jpg]
